# Computational Assessment of the Pharmacological Profiles of Degradation Products of Chitosan

**DOI:** 10.3389/fbioe.2019.00214

**Published:** 2019-09-06

**Authors:** Diana Larisa Roman, Marin Roman, Claudia Som, Mélanie Schmutz, Edgar Hernandez, Peter Wick, Tommaso Casalini, Giuseppe Perale, Vasile Ostafe, Adriana Isvoran

**Affiliations:** ^1^Advanced Environmental Research Laboratories, Department of Biology-Chemistry, Faculty of Chemistry, Biology, Geography, West University of Timisoara, Timisoara, Romania; ^2^Empa, Swiss Federal Laboratories for Materials Science and Technology, Technology and Society Laboratory, St. Gallen, Switzerland; ^3^Empa, Swiss Federal Laboratories for Materials Science and Technology, Particles-Biology Interactions Laboratory, St. Gallen, Switzerland; ^4^Department of Innovative Technologies, University of Applied Sciences and Arts of Southern Switzerland (SUPSI), Manno, Switzerland

**Keywords:** chito-oligomers, ADME-Tox, pharmacokinetics, toxicity endpoints, biological effects

## Abstract

Chitosan is a natural polymer revealing an increased potential to be used in different biomedical applications, including drug delivery systems, and tissue engineering. It implies the evaluation of the organism response to the biomaterial implantation. Low-molecular degradation products, the chito-oligomers, are resulting mainly from the influence of enzymes, which are found in the organism fluids. Within this study, we have performed the computational assessment of pharmacological profiles and toxicological effects on human health of small chito-oligomers with distinct molecular weights, deacetylation degrees, and acetylation patterns. Our approach is based on the fact that regulatory agencies and researchers in the drug development field rely on the use of modeling to predict biological effects and to guide decision making. To be considered as valid for regulatory purposes, every model that is used for predictions should be associated with a defined toxicological endpoint and has appropriate robustness and predictivity. Within this context, we have used FAF-Drugs4, SwissADME, and PreADMET tools to predict the oral bioavailability of chito-oligomers and SwissADME, PreADMET, and admetSAR2.0 tools to predict their pharmacokinetic profiles. The organs and genomic toxicities have been assessed using admetSAR2.0 and PreADMET tools but specific computational facilities have been also used for predicting different toxicological endpoints: Pred-Skin for skin sensitization, CarcinoPred-EL for carcinogenicity, Pred-hERG for cardiotoxicity, ENDOCRINE DISRUPTOME for endocrine disruption potential and Toxtree for carcinogenicity and mutagenicity. Our computational assessment showed that investigated chito-oligomers reflect promising pharmacological profiles and limited toxicological effects on humans, regardless of molecular weight, deacetylation degree, and acetylation pattern. According to our results, there is a possible inhibition of the organic anion transporting peptides OATP1B1 and/or OATP1B3, a weak potential of cardiotoxicity, a minor probability of affecting the androgen receptor, and phospholipidosis. Consequently, these results may be used to guide or to complement the existing *in vitro* and *in vivo* toxicity tests, to optimize biomaterials properties and to contribute to the selection of prototypes for nanocarriers.

## Introduction

Chitin is a polysaccharide abundantly found in nature, especially in crustaceans, but also in insects and fungi. Chitosan is obtained from chitin by chemical or enzymatic deacetylation (Rinaudo, 2006). The difference between chitin and chitosan consists of the acetyl content, chitin contains mostly N-acetyl-D-glucosamine (GlcNAc, A) units and chitosan contains especially D-glucosamine (GlcN, D). Chitin and chitosan reveal biocompatibility, biodegradability, and non-toxicity for humans and the environment (Rinaudo, 2006). These characteristics, added to the anti-fungal, anti-bacterial, anti-microbial, and anti-oxidant properties of chitosan conducted to numerous applications in different fields: food industry, cosmetic and personal care products, wastewater management, pharmacological products, and implantable materials (Enescu and Olteanu, 2008; Raafat and Sahl, 2009; Cheung et al., 2015). Chitosan nanoparticles are approved by the Food and Drug Administration (FDA) for tissue engineering and drug delivery and by FDA and EU for dietary use and wound dressing applications (Mohammed et al., 2017).

There are many different ways in which humans have exposure to chito-oligomers (COs): by the degradation of implanted materials based on chitosan, the use of pharmaceutical products containing COs and/or occupational exposure. The low-molecular degradation products of chitosan, the chito-oligomers, occur as a result of the influence of enzymes which are present in bodily fluids (Saikia et al., 2015). Due to the limitations concerning the applications of chitosan polymer related to its higher viscosity and insolubility in neutral and basic environments (Giri et al., 2012; Ways et al., 2018), COs with an increased solubility and lower viscosity have been obtained by chemical or enzymatic hydrolysis of chitosan and are largely used in the pharmaceutical field (Enescu and Olteanu, 2008; Patrulea et al., 2015; Ways et al., 2018). As an example, food supplements containing COs and their derivatives are used by many people for treating osteoarthritis (Jerosch, 2011) but their clinical importance is unclear (Liu et al., 2018). Occupational exposure to these compounds may also occur.

The chitosan oligomers that result from the hydrolysis processes may be classified in two types: (i) homo-chito-oligosaccharides containing only GlcN or GlcNAc units and (ii) hetero-chito-oligosaccharides, containing both GlcN and GlcNAc with varying degrees of deacetylation (the percent of glucosamine units in the oligomer, DDA) and a varying position of glucosamine residues in the oligomer chain (acetylation pattern, AP). GlcN unit carries an amino group that is protonated at physiological pH (Cheung et al., 2015). Both homo- and hetero-oligomers may differ in the degree of polymerization (the number the monomeric units within an oligomer, DP). Hetero-chito-oligomers with DP<10 are considered as water soluble (Liaqat and Eltem, 2018).

Taking into account the possible ways of exposure of humans to COs, the concept of Safe-by-design (SbD) must be considered when producing and using chitosan and its oligomers. Safe-by-design is a relatively new concept planned to be used in the research and development (R&D) field and industry, and include safe design, safe production, safe use and waste management for new materials and technologies (van de Poel and Robaey, 2017). Safe design addresses safety early in the R&D and design phases, such as in the design of safe products for professionals, consumers and the environment, and is usually based on prediction and experimental testing (*in vitro* and *in vivo* short term assays) tools. Safe production considers the use of environmentally friendly technologies and the potential risk for professionals involved in R&D and industrial processes. Safe use and waste management reflect safe handling (by both consumers and professionals) of products and wastes. Safety profiles of COs should depend on their structure and physicochemical properties (van de Poel and Robaey, 2017). Computational studies may have a valuable contribution to assess the safe designing of compounds by predicting the biological effects and toxicity profiles and by correlating them with the physicochemical and structural properties.

COs have numerous pharmaceutical properties and medical applications: anti-microbial, anti-oxidant, anti-tumoral, anti-inflammatory, immunostimulatory, anti-obesity, anti-hypertension, and anti-Alzheimer (Mourya et al., 2011; Cheung et al., 2015; Muanprasat and Chatsudthipong, 2017). COs having DP<20 are soluble in water and reveal antimicrobial, antioxidant, antitumoral, antiviral, antiangiogenic, and prebiotic properties (Sánchez et al., 2017; Long et al., 2018; Lu et al., 2019). COs with a molecular weight lower than 2000 Da and DDA>90% revealed a moderate neuroprotective activity and no toxicity against neurons (Santos-Moriano et al., 2018) and COs with molecular weight lower than 1000 Da showed high antioxidant activity (El-Sayed et al., 2017). Literature data reveal that COs having DP>6 possess enhanced anti-tumor, antimicrobial and immunopotentiation properties, favorable biological activities of smaller COs being also reported (Liaqat and Eltem, 2018). These activities of COs are dependent on their physicochemical properties: molecular weight (MW), the degree of deacetylation (DDA), the degree of polymerization (DP), and charge distribution (acetylation pattern, AP) (Park et al., 2011), but their structure–activity relationships are rather unknown (Santos-Moriano et al., 2018).

Specific literature contains little or no information about the biological effects of every possible variant of COs (Liaqat and Eltem, 2018). A chito-oligomer with specific physicochemical properties (MW, DDA, AP) may display all, some or none of all considered bioactivities of COs (Sánchez et al., 2017). The low reproducibility of results and sometimes the opposite reported effects concerning the COs biological activities could mainly be due to relatively poorly characterized oligomer mixtures that have been used in experimental studies and/or to inconstant reporting of the properties of COs (Mourya et al., 2011; Park et al., 2011; Li et al., 2016). Furthermore, available information concerning the effects of COs is mainly derived from *in vitro* and *in vivo* studies with animal models and there are limited human experimental data concerning the absorption, distribution, metabolism, excretion, and toxicity of these compounds (Cheung et al., 2015; Phil et al., 2018). It reflects the insufficiency of safety data concerning their use by humans.

The number of used chemicals is increasing constantly and there is a need to assess their safety for humans and environment. Considering the costs of the laboratory studies and the ethical concerns on using animals for testing, the role of bioinformatics in hazard assessment is well-recognized. The explosive growth in the magnitude and diversity of data from physics, chemistry, and biology conducted to the creation of specific databases and computational packages for data manipulation (Luechtefeld and Hartung, 2017). The easy access to these data allowed scientists to build accurate computational models for toxicology assessment. These models are used in drug discovery and development and for assessment of the effects of xenobiotics on humans and environment. Computational tools that were developed for hazard assessment include (quantitative) structure-activity relationships [(Q)SARs], read-across methods, expert rule-based (structural alerts) methods, and molecular modeling techniques (Alves et al., 2018a; Myatt et al., 2018; Yang et al., 2018). The Organization of Economic and Co-operation Development (OECD) created QSAR guidelines already in 2004 and the principles for the construction of (Q)SAR models, computational methods, and model validation methods are described in detail since 2007 (Fjodorova et al., 2008; Lo Piparo and Worth, 2010). Furthermore, REACH (Registration, Evaluation and Authorization of CHemicals) regulation mentions the QSAR techniques for studying the toxicological profile of chemicals (Kleandrova and Speck-Planche, 2013). Consequently, QSAR has been a reliable computational tool used for decades for connecting the properties and biological activity. Taking into consideration the limitations, the number of improvements has been recorded and various descriptors have been explored: molecular properties (0D-QSAR), fragment counts (1D-QSAR), topological descriptors (2D-QSAR), spatial coordinates (3D-QSAR), a combination of atomic coordinates and sampling of conformations (4D-QSAR), multiple expression of ligand topology (5D-QSAR), considering the solvation function (6D-QSAR) and receptor or target-based receptor model data (7D-QSR) (Kar and Leszczynski, 2019). Nowadays, many software and web servers are available for predicting chemical toxicity before synthesis, as it is recognized that computational techniques provide high-quality predictions for chemical hazard assessment (Yang et al., 2018), meaning 2D-QSAR and 3D-QSAR methods are frequently used.

Besides the large applicability of these modern tools for human and environment hazard assessment, there also are some limitations, mostly related to the robustness and predictability of the used models and to the fact that they do not provide a clean mechanistic interpretation of the outcomes (Luechtefeld and Hartung, 2017). The methods mentioned above are not applicable for assessing the pharmacokinetics of nanoparticles, especially due to the fact that fundamental mechanisms that support drug-handling within the human organism are not understood for nanoparticles (Nel et al., 2009; Beddoes et al., 2015). Also, as most QSAR models are based on *in vivo* or *in vitro* data from specific experimental conditions, the applicability domain of the QSAR model is more limited for nanomaterials (Choi et al., 2018). Furthermore, data concerning the effects of the oligomer components cannot be transferred to nanoscale polymers since in the case of nanoparticles, not only the dose and their elemental composition, but their shape, size, and surface characteristics determine the biological activities and therapeutic effects and it increases the difficulty of modeling the biological effects of nanomaterials (Nel et al., 2009; Beddoes et al., 2015). Consequently, within this study we focus on the chito-oligomers both as degradation products of chitosan nanoparticles and as independent food supplements.

The objectives of this study are: (1) prediction of the pharmacological profiles and toxicological endpoints (skin sensitization potential, endocrine disruption potential, cardiotoxicity, hERG channel blocking potential, carcinogenicity, and mutagenicity) of COs containing up to 8 monomeric units (water soluble chito-oligomers) and (2) assessment of the influence of the MW, DDA, and AP on the toxicological and pharmacological profiles of investigated COs by using computational approaches.

## Materials and Methods

Among the numerous available computational tools for predicting the pharmacological properties and toxicological effects of chemical compounds on human health, we have selected those with an accuracy of a prediction usually higher than 70% and with friendly interfaces and tutorials that are available for free (online or open-source). The chito-oligomers that we have considered in this study are presented in Table 1 together with the computed values of their molecular weights using admetSAR2.0 tool (see further). We specify that each amino group of the deacetylated units is protonated. Furthermore, Table 1 shortly reviews known information concerning medical and side/toxicological effects of small COs. Some of these compounds are approved by FDA only as food supplements and/or for use in wound dressings (Wedmore et al., 2000). In Europe, GlcN and GlcNAc are approved as drugs in the form of glucosamine sulfate (Jordan et al., 2003).

**Table 1:** Chito-oligomers considered in this study, their computed molecular weights (MW), and known medical and side effects (NA means not available data).

**DD**	**Acetylation pattern**	**MW (g/mol)**	**Medical effects**	**Side effects**
0%	A	221.21	N-acetyl-D-glucosamine is used in treating osteoarthritis, cancer, and wounds (Jordan et al., 2003; Masuda et al., 2014). It proved to be useful for treating colds and pain (Konno, 2002) and is found in cosmetic products being able to reduce the facial hyperpigmentation (Bissett et al., 2007).	A study concerning oral administration of GlcNAc at doses of 500 and 1000 mg/day for 68 female revealed no side effects (Kubomura et al., 2017).
	2A 3A	424.40 627.59	Di-N-acetyl chitobiose and tri-N-acetyl chitotriose did not show anti-oxidant activity *in vitro* (Chen et al., 2003) but proved to be useful for treating colds and pain (Konno, 2002).	NA
	4A 5A	870.79 1,033.98	Tetra-N acetyl-chitotetraose and penta N-acetyl chitopentaose have been used for treating colds and pain (Konno, 2002). Tetra-N acetyl-chitotetraose significantly improved both learning and of rats suffering of Alzheimer's disease (Jiang et al., 2019).	NA
	6A	1,237.17	Hexa N -acetyl chitohexaose revealed a tumor grows inhibitory effect (Xiong et al., 2009) and had favorable influence in treating colds and pain (Konno, 2002). Chitohexaose blocks the induction of inflammatory mediators both *in vitro* and *in vivo* (Das et al., 2019) and significantly improved both learning and of rats suffering of Alzheimer's disease (Jiang et al., 2019).	NA
	8A	1,643.56	Octa N -acetyl chitooctose had favorable influence in treating colds and pain (Konno, 2002).	NA
33%	ADA	585.56	N,N′-diacetylchitotriose exhibited an anti-oxidant activity *in vitro* (Li et al., 2013).	NA
50%	DA	382.36	NA	NA
	DADA	746.71	NA	NA
	ADAD	746.71	NA	NA
	AADD	746.71	NA	NA
	DDAA	746.71	NA	NA
	DAAD	746.71	NA	NA
	ADDA	746.71	NA	NA
	DADADA	1,475.41	NA	NA
	ADADAD	1,475.41	NA	NA
	DADADADA	1,857.77	NA	NA
67%	DDA	543.52	N-acetylchitotriose revealed an anti-oxidant activity *in vitro* (Li et al., 2013).	NA
	ADDDAD	1,069.02	NA	NA
	DDDADA	1,069.02	NA	NA
100%	D	179.17	Glucosamine is a popular food supplement used for treating osteoarthritis, but clinical trials on humans did not reveal results supporting its efficacy for every human subject (Chan and Fat, 2011; Liu et al., 2018). Glucosamine administration is expected to promote wound healing by enhancing hyaluronic acid production (Esfahani et al., 2012). It has anti-inflammatory, anti-aging, anti-oxidant, anti-cancer, anti-fibrotic, anti-fungal, neuro-protective, cardio-protective, skin hydration, and wrinkle reduction properties (Masuda et al., 2014; Zahedipour et al., 2017; Fawzya et al., 2019). It induced weight loss and reduced triglyceride and cholesterol levels in serum (Huang et al., 2015).	Clinical trial data obtained for 3063 human subjects revealed non effects of the oral administration of glucosamine on glucose metabolism and on urine, blood, and fecal parameters (Anderson et al., 2005). It may induce mild gastrointestinal disorders (Dalirfardouei et al., 2016).
	2D	340.33	Chitobiose had a strong anti-oxidant activity *in* (Chen et al., 2003) and has been used for treating common colds and pain (Konno, 2002). Chitobiose revealed a significant inhibitory effect on hepatic lipid accumulation *in vitro* (Li et al., 2018; Zhao et al., 2019) and anti-bacterial effect on Gram-positive bacteria (Li et al., 2014).	NA
	3D	501.48	Chitotriose revealed potency to treat colds and pain (Konno, 2002), strong anti-oxidant activity *in vitro* (Chen et al., 2003), a low inhibitory effect on hepatic lipid accumulation *in vitro* (Li et al., 2018; Zhao et al., 2019) and an anti-bacterial effect on Gram-positive bacteria (Li et al., 2014). Chitotriose seems to have beneficial effects on the nervous system (Jiang et al., 2014).	NA
	4D	662.64	Chitotetraose revealed a low inhibitory effect on hepatic lipid accumulation *in vitro* (Li et al., 2018) and an anti-bacterial effect on Gram-positive bacteria (Li et al., 2014). It had favorable properties for treating pain and colds (Konno, 2002).	NA
	5D	823.79	Chitopentaose revealed a low inhibitory effect on hepatic lipid accumulation *in vitro* (Li et al., 2018), an enhanced anti-bacterial effect on Gram-positive bacteria (Li et al., 2014) and anti-inflammatory action *in vitro* (Li et al., 2012).	NA
	6D	984.95	Chitohexaose revealed a low inhibitory effect on hepatic lipid accumulation *in vitro* (Li et al., 2018), exhibited a high anti-tumor activity *in vitro* (Xiong et al., 2009; Li et al., 2016) and anti-fungal activity (Fawzya et al., 2019).	NA
	8D	1,307.26	Chitooctose had favorable influence in treating colds and pain (Konno, 2002).	NA

The simplified molecular-input line-entry system (SMILES) formulas of the considered COs are built using ACD/ChemSketch software (https://chemicalize.com). This software also generates structural files in *mol* format to be used for further predictions We have obtained 3D sdf files using OpenBabel (O'Boyle et al., 2011) on the online server http://www.cheminfo.org/Chemistry/Cheminformatics/FormatConverter/index.html, starting from their structural files in *mol* format generated by ACD/ChemSketch software. Structure minimization has been done using Chimera software (Pettersen et al., 2004) using 1000 steepest descent steps, step size 0.02 Å, 10 conjugate gradient steps, conjugate gradient step size 0.02 Å.

FAF-Drugs4 (Lagorce et al., 2017) tool has been considered for assessing the oral bioavailability as a part of the pharmacokinetic profile and the overall toxicity of investigated COs. This is a rule-based tool having the accuracy of predictions higher than 70% (Lagorce et al., 2017). FAF-Drugs4 tool allows filtering against Lipinski's rule (Lipinski et al., 2001), Egan's rule (Egan et al., 2000), and Veber's rule (Veber et al., 2002) for predicting bioavailability and of Pfizer's and GSK rules for predicting the overall toxicity (Gleeson, 2008).

SwissADME is a web tool that allows the computation of the physicochemical parameters of a chemical compound, its pharmacokinetic profile, drug likeness and medicinal chemistry, starting from the SMILES formula, the accuracy of predictions being between 72 and 94% (Daina et al., 2017).

AdmetSAR2.0 (Cheng et al., 2012; Yang et al., 2019) tool has been used to predict pharmacokinetic profiles and organ (eye, heart, liver) and genomic toxicity of investigated COs. with a predictive accuracy of 72.3–76.7% (Yang et al., 2019). Furthermore, every prediction made by this tool is quantitatively described by a probability output.

PreADMET is also a web tool having four parts: (i) molecular descriptors calculation; (ii) drug likeness prediction considering well known rules; (iii) ADME prediction; and (iv) toxicity prediction [mutagenicity by Ames test and rodent carcinogenicity; (Lee et al., 2003, 2004)].

Because occupational exposure to chitin and chitosan may also occur through dermal contact and skin sensitization, it may have a significant impact on individual working capacity and quality of life, we have assessed the skin sensitizer potential of investigated COs using Pred-Skin computational tool (Braga et al., 2017; Alves et al., 2018b). This information is also important when we take into account the fact that chitosan is approved to be used in wound healing purposes. Pred-Skin is a web-based computational facility considering QSAR models of skin sensitization potential. It performs the following predictions: (i) binary predictions of human skin sensitization potential established taking into account human data (prediction accuracy being 73–76%); (ii) binary predictions of murine skin sensitization potential taking into account animal data (LLNA, prediction accuracy being 70–84%); (iii) binary predictions based on Direct Peptide Reactivity Assay (DPRA), KeratinoSens, and the human Cell Line Activation Test (h-CLAT) data (prediction accuracy being 80–86%); (iv) a consensus model that is generated by averaging the predictions of individual models (prediction accuracy being 70–84% (Braga et al., 2017; Alves et al., 2018b).

Predictions concerning carcinogenicity and mutagenicity are also obtained using Toxtree software, the accuracy of predictions being 70% (Patlewicz et al., 2008).

CarcinoPred-EL (Carcinogenicity Prediction using Ensemble Learning methods) utility has been used for accomplishing predictions concerning the carcinogenicity of investigated chemicals (Zhang et al., 2017). It is a free prediction online server that is based on twelve different molecular fingerprints and three ensemble machine learning models (Ensemble RF, Ensemble SVM, and Ensemble XGBoost) permitting the identification of the structural features related to carcinogenic effects of chemical compounds (Zhang et al., 2017).

Pred-hERG is another free accessible web tool that builds predictive models of the ability of a chemical compound to inhibit the human ether-à-go-go related gene (hERG) K^+^ channels. This hERG K^+^ channel blockage may result in cardiac side effects such as heart arrhythmia and even possibly death (Braga et al., 2015). Consequently, hERG K^+^ channel blockage is one of the most important toxicological endpoints to be considered when assessing the safety of chemical compounds. There are two outcomes when using Pred-hERG tool: a binary prediction (hERG non-blocker or blocker) and a multiclass prediction (hERG non-blocker, weak/moderate blocker, strong blocker) along with the probability of the prediction for each class. The predictions have an accuracy of up to 89% (Braga et al., 2015).

Endocrine disruption potential is evaluated using ENDOCRINE DISRUPTOME computational tool (Kolšek et al., 2014). This tool uses the molecular docking approach for predicting interactions between the explored compound with 12 distinct human nuclear receptors, those binding sites are known: androgen receptor (AR), estrogen receptors α (ERα) and β (ERβ), glucocorticoid receptor (GR), liver X receptors α (LXRα) and β (LRXβ), peroxisome proliferator-activated receptors α (PPRAα), β/δ (PPRAβ), and γ (PPRAγ), retinoid X receptor α (RXRα) and thyroid receptors α (TRα), and β (TRβ). Both agonistic and antagonistic (an) effects are predicted for AR, ERα, ERβ, and GR. Predictions are based on computation of the sensitivity (SE) parameter and compounds are categorized in four classes: (i) compounds with SE<0.25 expose a high probability of binding to nuclear receptors; (ii) compounds with 0.25<SE<0.50 reflect a medium probability of binding to the nuclear receptors; (iii) compounds having 0.50<SE<0.75 emphasize minor probability of binding and (iv) compounds with SE>0.75 reveal a low probability of binding to the nuclear receptors (Kolšek et al., 2014).

A summary of the computational tools that we have used in this study is presented in Table 2.

**Table 2:** Short presentation of the computational tools that were used in the current study.

**Tool**	**Inputs**	**Method**	**Output**	**References**
FAF-Drugs4	Structural data files (2D SDF) of COs	expert-rules based	Oral bioavailability and safety profiles	Lagorce et al., 2017
SwissADME	SMILES formulas of COs	expert-rules based 2D QSAR	Druglikeness Pharmacokinetic profile	Daina et al., 2017
PreADMET	Structural data files (2D SDF) of COs	expert-rules based 2D-QSAR	Druglikeness Pharmacokinetic profile Toxicological endpoint	Lee et al., 2003, 2004
admetSAR2.0	SMILES formulas of COs	2D QSAR	Pharmacokinetic profiles, organ (eye, heart, liver) and genomic toxicity	Cheng et al., 2012; Yang et al., 2019
Pred-Skin	SMILES formulas of COs	2D-QSAR	Skin sensitization potential	Braga et al., 2017; Alves et al., 2018b
Toxtree	SMILES formulas of COs	Expert-rules based	Carcinogenic and mutagenic potential	Patlewicz et al., 2008
CarcinoPred-EL	SMILES formulas of COs	2D QSAR	Carcinogenic potential	Zhang et al., 2017
Pred-hERG	SMILES formulas of COs	2D QSAR	hERG K+ channel blockage potential	Braga et al., 2015
ENDOCRINE DISRUPTOME	SMILES formulas of COs	Molecular docking and calculation of a sensitivity parameter	Probability of binding to nuclear receptors	Kolšek et al., 2014

The computational tools that are used in this study have been elaborated for assessing the pharmacological profiles and toxicological endpoints of new drugs, but they were successfully applied for other classes of chemicals: cosmetic ingredients and pesticides (Alves et al., 2018c; Roman et al., 2018a; Gridan et al., 2019), synthetic steroids found on the market as food supplements or veterinary drugs (Roman et al., 2018b), water soluble derivatives of chitosan (Isvoran et al., 2017). It illustrates their applicability for predicting pharmacological properties and toxicological endpoints for many classes of compounds.

## Results

Estimation of the oral bioavailability and overall toxicity of investigated COs is obtained using FAF-Drugs4 tool and is based on filtering the physicochemical properties of investigated compounds in accordance with the rules mentioned above. FAF-Drugs4 tool also estimates if the investigated compounds are able to produce phospholipidosis (PI). The outcomes are presented in Table 3.

**Table 3:** Estimation of oral bioavailability and overall toxicity of chito-oligomers: green cells correspond to respected rules (0 violations), yellow cells correspond to partially respected rules (maximum 2 violations for Lipinski's rule and 1 violation for Veber's and Eagan's rules), light red cells correspond to broken rules.

**Compound**	**Oral bioavailability**			**Overall toxicity**		
	**Lipinski's rule**	**Veber's rule**	**Eagan's rule**	**Pfizer's rule**	**GSK rule**	**PI**
A						No
2A	2 violations HBA>10 HBD>5					No
3A, 4A, 5A, 6A, 8A	3 violations MW>500 HBA>10 HBD>5					No
ADA, DAD	3 violations MW>500 HBA>10 HBD>5					Yes
AD	2 violations HBA>10 HBD>5					Yes
ADAD, DADA, DAAD, DDAA, AADD ADDA ADADAD, DADADA DDDADA ADDDAD, DADADADA	3 violations MW>500 HBA>10 HBD>5					Yes
D	2 violations HBD>5, HBA>5					Yes
2D, 3D, 4D, 5D, 6D, 8D	3 violations MW>500 HBA>10 HBD>5					Yes

*The number of violation for every considered rule is specified. Compounds expected to not induce phospholipidosis (PI) are marked by “No” in green cells and compounds expected to induce phospholipidosis are marked by “yes” in light red cells. (MW-molecular weight, HBA – hydrogen bond acceptors, HBD- hydrogen bonds donors)*.

**Figure 1:**
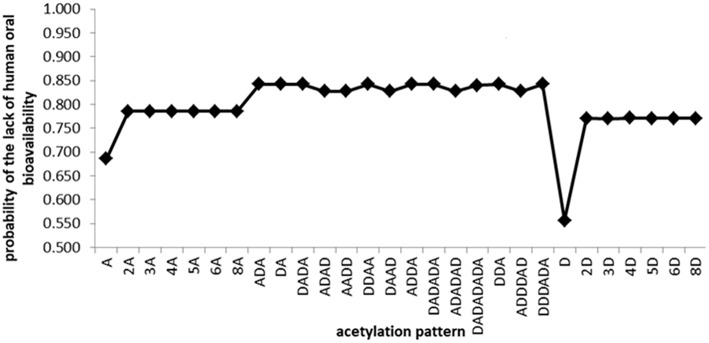
Predictions obtained using admetSAR2.0 tool concerning the lack of the human oral bioavailability of investigated chito-oligomers. The predicted probabilities of the lack of oral bioavailability may take values between 0 and 1. As the value is closer to 1, the oral bioavailability is missing.

With the exceptions of the monomeric and some of the dimeric chito-oligomers, the outcomes of FAF-Drugs4 indicate the lack of oral bioavailability of the other investigated COs because of their molecular weight and extensive hydrogen bonding potential. Similar results concerning the lack of human oral bioavailability of investigated COs containing more than 2 monomeric units have been obtained using admetSAR2.0 (Figure 1), PreADMET and SwissADME tools (Supplementary Table 1).

As expected, the oral bioavailability decreases with increasing molecular weight and increases with the deacetylation degree. PreADMET predictions concerning the percent of the human intestinal absorption (HIA, Supplementary Table 2) reveal a mean absorbance (20%<HIA<70%) (Aswathy et al., 2018) for the monomeric units, the highest value (60.25%) being registered for the GlcN oligomer. Chito-oligomers containing two monomeric units reflect a poor absorption (HIA<20%) and the other COs do not reflect intestinal absorption (HIA = 0). Predictions obtained using SwissADME tool reveal low gastrointestinal absorption (GI) for all investigated oligomers (Supplementary Table 3). All of these results suggest that smaller and deacetylated COs could be better absorbed at the gastrointestinal level and it facilitates their access to systemic circulation and distribution through the human body.

**Figure 2:**
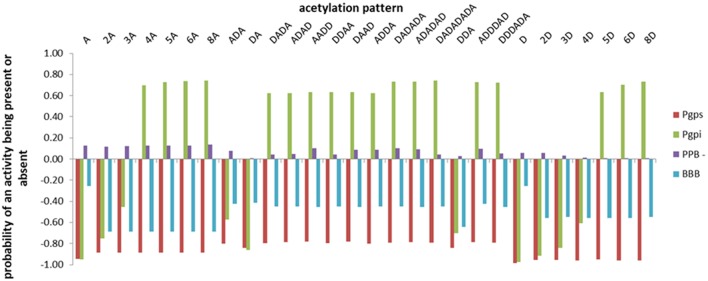
The distribution profiles of the investigated chito-oligomers expressed as the probabilities of binding to plasma proteins (PPB), being substrate/inhibitor of the P-glycoprotein (P-gpS/P-gpI), being able to penetrate the blood-brain barrier (BBB). The predicted probabilities may take values between 0 and 1 when the investigated activity is present and between −1 and 0 when the activity is considered absent. Values closer to 1 correspond to effects that are highly probable and values closer to −1 correspond to highly improbable effects.

Predictions concerning the distribution (expressed as the probability of plasma protein binding—PPB, being P-glycoprotein substrate and/or inhibitor, being able to penetrate the blood-brain barrier– BBB) of the investigated COs have been obtained using admetSAR2.0 tool and the outcomes are illustrated in Figure 2. Negative values of the probabilities illustrate that the investigated activity is absent.

Data presented in Figure 2 illustrate that investigated COs reveal a very low probability to bind to plasma proteins, they are not able to penetrate the blood brain barrier and to affect the central nervous system, oligomers with more than 3 monomeric units reflect a small probability to inhibit the P-glycoprotein and none of the investigated COs is considered as P-glycoprotein substrate. There are small differences in the values of predicted probabilities for a given activity between oligomers with the same DP and different DDA, reflecting the influence of the deacetylation degree on the activity of chito-oligomers. Almost similar predictions are obtained using PreADMET (Supplementary Table 2) and SwissADME (Supplementary Table 3) tools. PreADMET reveals that investigated COs are not inhibitors of the P-glycoprotein and outcomes values for the blood brain barrier penetration that are lower than 0.1 that correspond to a low absorbance to central nervous system (Aswathy et al., 2018). The PPB binding assessment (percentage of drug bound in plasma protein) reveals that investigated COs exhibit low binding energy with plasma proteins (PPB<90%) (Kandagalla et al., 2017) with the exception of the oligomer 6D that shows a high binding energy to plasma proteins (93.384%). SwissADME predicts that investigated compounds are not able to penetrate the blood brain barrier and are considered as substrates of P-glycoprotein.

SwissADME, PreADMET, and admetSAR2.0 tools have been also used to assess the metabolism of COs by predicting the probability for every compound to be a substrate or to inhibit the human cytochromes P450 (CYP) involved in the metabolism of xenobiotics. The outcomes of SwissADME and admetSAR2.0 tools indicate that COs considered in this study are not substrates and inhibitors of CYPs (Supplementary Tables 3, 4). The predictions obtained using PreADMET are not similar, they indicate that investigated COs are not considered as substrates and inhibitors of CYP2C9 and CYP2C19, but the oligomers containing deacetylated units are possible dual inhibitors and substrates of CYP2D6 and CYP3A4. It may be due to complex modulation of CYP enzymes and this aspect should be further analyzed.

**Figure 3:**
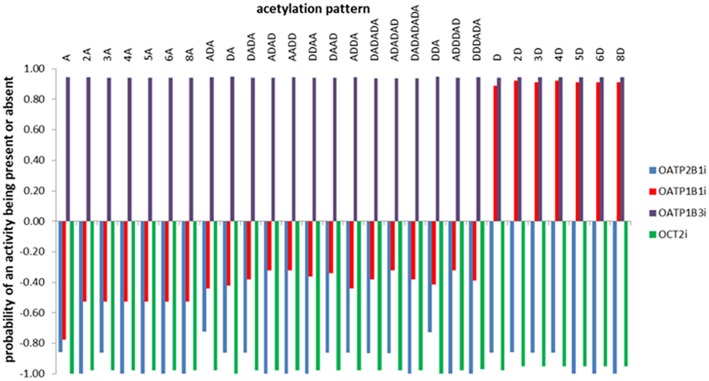
Predictions concerning the probability of the inhibition of the organic anion and cation transporter peptides by the investigated COs. The predicted probabilities may take values between 0 and 1 when the investigated activity is present, and between −1 and 0 when the activity is considered absent. Values closer to 1 correspond to effects that are highly probable and values closer to −1 correspond to highly improbable effects.

Predictions concerning the probability of the inhibition of the organic anion and cation transporter peptides by the investigated COs are also obtained using admetSAR2.0 tool and are illustrated in Figure 3. This figure suggests the inhibitory potential of all investigated COs against organic anion polypeptide transporter OATP1B3. Deacetylated oligomers also illustrate the inhibitory potential against OATP1B1 and underline the influence of the DDA on the activity of investigated COs.

Predictions concerning the organ (eye, heart, liver) and genomic toxicity of investigated COs obtained using admetSAR2.0 and PreADMET tools are revealed in Table 4.

**Table 4:** Predictions obtained using admetSAR and PreADMET tools concerning the probabilities of organ and genomic toxicity of investigated COs: hERG – potassium channel blocking potential (cardiotoxicity), EC- eye corrosion, EI – eye irritation, HEPT – hepatotoxicity.

**Compound/tool**	**admetSAR2.0**	**Pre-ADMET**	**admet SAR2.0**	**Pre-ADMET**	**admetSAR2.0**	**Pre-ADMET**	**admetSAR2.0**		
	**hERG**		**Ames mutagenesis**		**Mouse carcinogenicity**	**Mouse and rat carcinogenicity**	**EC**	**EI**	**HEPT**
A	−0.62	low_risk	−0.54	Mutagen	−0.94	Negative	−0.99	−1.00	−0.65
2A	−0.44	Low_risk	−0.57	Mutagen	−0.96	negative	−0.99	−0.97	−0.58
3A	0.76	Ambiguous	−0.57	Mutagen	−0.96	Negative	−0.99	−0.92	0.63
4A	0.79	Ambiguous	−0.57	Mutagen	−0.96	Negative	−0.99	−0.90	0.78
5A	0.80	Ambiguous	−0.57	Mutagen	−0.96	Negative	−0.99	−0.90	0.78
6A	0.80	Ambiguous	−0.57	Mutagen	−0.96	Negative	−0.99	−0.90	−0.50
8A	0.80	Ambiguous	−0.57	Mutagen	−0.96	Negative	−0.99	−0.90	−0.60
ADA	0.73	Ambiguous	−0.51	Non-mutagen	−0.96	Negative	−0.99	−0.94	0.58
DA	−0.49	Low_risk	−0.57	Mutagen	−0.96	negative	−0.99	−0.99	−0.55
DADA	0.86	Ambiguous	−0.56	Non-mutagen	−0.96	Negative	−0.99	−0.92	0.65
ADAD	0.77	Ambiguous	−0.52	Non-mutagen	−0.96	Negative	−0.99	−0.92	0.68
AADD	0.79	ambiguous	−0.51	Non-mutagen	−0.95	Negative	−0.99	−0.92	0.68
DDAA	0.79	Ambiguous	−0.56	Non-mutagen	−0.96	Negative	−0.99	−0.91	0.70
DAAD	0.80	Ambiguous	−0.59	Non-mutagen	−0.96	Negative	−0.99	−0.91	0.72
ADDA	0.76	Ambiguous	−0.51	non-mutagen	−0.95	Negative	−0.99	−0.91	0.70
DADADA	0.82	Ambiguous	−0.56	Non-mutagen	−0.96	Negative	−0.99	−0.90	0.60
ADADAD	0.81	Ambiguous	−0.51	Non-mutagen	−0.96	Negative	−0.99	−0.90	0.55
DADADADA	0.77	Ambiguous	−0.61	Non-mutagen	−0.95	Negative	−0.99	−0.90	−0.56
DDA	0.68	Ambiguous	−0.57	Mutagen	−0.96	Negative	−0.99	−0.96	−0.50
ADDDAD	0.82	Ambiguous	−0.52	Non-mutagen	−0.95	Negative	−0.99	−0.90	0.58
DDDADA	0.81	Ambiguous	−0.56	Non-mutagen	−0.95	Negative	−0.99	−0.90	0.55
D	−0.67	Low_risk	−0.70	Mutagen	−0.97	Negative	−0.99	−0.99	−0.90
2D	−0.43	low_risk	−0.71	Mutagen	−0.97	Negative	−0.99	−0.98	−0.95
3D	0.72	Ambiguous	−0.71	Mutagen	−0.97	Negative	−0.99	−0.94	−0.85
4D	0.73	Ambiguous	−0.71	Non-mutagen	−0.99	Negative	−0.99	−0.92	−0.68
5D	0.73	Ambiguous	−0.71	Non-mutagen	−0.99	Negative	−0.99	−0.90	0.53
6D	0.82	Ambiguous	−0.71	Non-mutagen	−0.97	Negative	−0.99	−0.90	0.53
8D	0.83	Too big to be computed	−0.71	Too big to be computed	−0.97	Too big to be computed	−0.99	−0.90	−0.50

*Negative values of the probabilities illustrate that the investigated activity is absent. These probabilities may take values between −1 and 0 when the predicted activity is absent and between 0 and 1.00 when the predicted activity is present*.

None of the investigated oligomers have carcinogenic potential, does not produce eye irritations and corrosion. Excepting the monomeric and dimeric units that are not predicted to block the hERG, the other oligomers reveal moderate potentials of hERG channel blocking. Totally acetylated oligomers and small oligomers containing deacetylated units are predicted by the PreADMET tool as displaying mutagenic potential. Besides admetSAR2.0 and PreADMET tools, carcinogenicity and mutagenicity of investigated COs have also been assessed using CarcinoPred-EL and Toxtree and their outcomes displayed no carcinogenicity and no Ames toxicity for every considered oligomer (Supplementary Table 5). The consensus of the predictions made by the majority of these computational tools emphasizes that none of the investigated COs is expected to be carcinogen and mutagen. Data presented in Table 4 reveal different values for the predicted probabilities for COs with distinct DDA and AP and it underlines the dependence of the biological activities of investigated COs on their properties, DDA, and AP.

For assessing cardiotoxicity of investigated COs we have also considered Pred-hERG prediction tool and the results are presented in Table 5.

**Table 5:** Outcomes of the Pred-hERG computational tool concerning the blockage of the potassium channel by the investigated chito-oligomers: red cells illustrate predictions of hERG blocking potential and green cells illustrate hERG non-blocking potential.

**Compound**	**Pred-hERG**	
	**Prediction by binary model**	**Prediction by multiclass model non-blocker**
A	0.5	0.8
2A	0.6	0.7
3A	0.6	0.7
4A	0.6	0.7
5A	0.6	0.7
6A	0.6	0.7
8A	0.6	0.7
ADA	0.7	0.7
DA	0.6	0.7
ADAD	0.7	0.7
DADA	0.7	0.7
ADDA	0.7	0.7
AADD	0.7	0.7
DAAD	0.7	0.7
DDAA	0.7	0.7
ADADAD	0.7	0.7
DADADA	0.7	0.7
DADADADA	0.7	0.7
DDA	0.7	0.7
ADDDAD	0.7	0.7
DDDADA	0.7	0.7
D	0.5	0.8
2D	0.6	0.7
3D	0.6	0.7
4D	0.6	0.7
5D	0.6	0.7
6D	0.7	0.7
8D	0.6	0.7

*Number in every cell represents the probability of the prediction for each class. The values vary between 0 and 1*.

Predictions made using the binary model illustrate that, except GlcN and GlcNAc monomers, that are considered as non-hERG K+ blockers, the other investigated COs are predicted as having hERG K+ blocking potential but the probabilities of these predictions are relatively small. Predictions based on the multiclass models reveal non-hERG blocking potential for all investigated COs, also with relatively small probabilities. Predictions reliability reported using Pred-hERG are ranging between 83 and 84% for the binary model and between 66 and 79% for the multiclass models (Braga et al., 2015). Consequently, we deliberate that, excepting the monomers, the other investigated COs reveal a weak hERG K+ blocking potential, this outcome being in very good correlation with predictions made by admetSAR2.0 and PreADMET tools (see Table 4).

The use of Pred-Skin computational tool reveals that investigated oligomers reflect no skin sensitizer potential (Supplementary Table 6). It is an important result as skin sensitization is known to be a common occupational health issue (Anderson and Meade, 2014). SwissADME and PreADMET tools also predicted very small values of the skin permeability parameters (Supplementary Tables 2, 3). The value of skin permeability parameter computed using SwissADME tool for diclofenac (an anti-inflammatory drug known to permeate the skin) is logKp = −4.96 cm/s (Daina et al., 2017). The use of PreADMET tool to compute the skin permeability coefficient for betulinic acid conducted to the value of logKp = −2.11 cm/h proving that betulinic acid is not permeable through the skin (Khan et al., 2018). The values of logKp computed for investigated COs by using SwissADME and PreADMET tools are much smaller that the two indicated values and it illustrates that these compounds are not permeable through skin.

Endocrine disruption potential is another toxicological endpoint that must be considered when using chemical compounds. Assessment of the endocrine disruption potential of investigated COs has been obtained using ENDOCRINE DISRUPTOME prediction tool and the results are presented in Table 6. Monomers and dimers of GlcNAc and GlcN, and chitobiose (DA) have the moderate ability (0.75>SE>0.50, yellow cells in Table 6) to bind to the androgen receptor and to produce antagonistic effects. The dimers AA and DA and the trimers ADA and DDD reflect a moderate potential to bind to the glucocorticoid receptor and to produce agonistic effects (0.75>SE>0.50, yellow cells in Table 6).

**Table 6:** Outcomes of the ENDOCRINE DISRUPTOME prediction tool concerning the potential binding of investigated COs to the human nuclear receptors: androgen receptor (AR), estrogen receptors α (ERα) and β (ERβ), glucocorticoid receptor (GR), liver X receptors α (LXRα), and β (LRXβ), peroxisome proliferator-activated receptors α (PPRAα), β/δ (PPRAβ), and γ (PPRAγ), retinoid X receptor α (RXRα) and thyroid receptors α (TRα) and β (TRβ), an - antagonistic effect.

**Acetylation pattern**	**AR**	**AR an**	**ERα**	**ERα an**	**ERβ**	**ERβ an**	**GR**	**GR an**	**LXRα**	**LXRβ**	**PPARα**	**PPARβ**	**PPARγ**	**RXR α**	**TRα**	**TRβ**
A																
AA																
AAA																
DA																
ADA																
DADA																
AADD																
DAAD																
DDAA																
ADDA																
DDA																
ADAD																
D																
DD																
DDD																
DDDD																

*Results are color coded taking into account the values of the sensitivity parameter. Class “yellow” corresponds to 0.50<SE<0.75 and indicates compounds with moderate probability of binding, and class “green” corresponds to SE>0.75 and illustrates compounds with low probability of binding to the nuclear receptors*.

It means that smaller COs may inhibit the androgen and the glucocorticoid receptors and could be capable of deleterious effects on the male reproductive tract or to affect the immune response of the organism. COs containing more than 4 monomers are too big to accommodate in the binding site of the human nuclear receptors considered by the ENDOCRINE DISRUPTOME prediction tool and there are not outcomes, calculations being aborted. It seems that molecular weight is an important property for the COs that are able to interact with nuclear receptors. This outcome is in good agreement with literature data revealing that small organic non-steroidal molecules (MW between 430 and 600 Da) are capable to act as AR antagonists (Song et al., 2012; Tesei et al., 2013) and to interact with GR (Harcken et al., 2014; Sundahl et al., 2015).

## Discussion

The outcomes of the computational tools have been used to study the chito-oligomers pharmacological profiles and their toxicity. It has been shown that the results obtained with the different computational tools are usually in accordance with each other. The results obtained using FAF-Drugs4, admetSAR2.0, and PreADMET reveal that oligomers GlcNAc, GlcN, and GlcNAc-GlcN may have a higher oral bioavailability by comparison to the other COs, showing that oral bioavailability seems to be decreasing with increasing molecular weight. This outcome is in good correlation with published data considering that chitosan's systemic absorption and distribution is dependent on the molecular weight, oligomers could reveal some absorption whereas larger polymers are excreted (Kean and Thanou, 2010). Other studies revealed that the absorption of chito-oligomers was significantly influenced by the molecular weight, the absorption decreased with increasing molecular weight (Chae et al., 2005; Naveed et al., 2019). Moreover, low molecular weight COs produced by depolymerization are usually preferred for pharmaceutical applications (Quiñones et al., 2018) as they have been reported to show remarkable biological activities (Adhikari and Yadav, 2018).

The action of chemicals depends on their interactions with plasma proteins, with unbound molecules usually reflecting better interactions with their targets and influencing the efficacy of the molecules (Kandagalla et al., 2017). All computational facilities that we have used reveal that COs considered in this study exhibit low potential to bind to plasma proteins. Consequently, these compounds exist freely being available for transport across the cell membranes, for the interaction with specific/non-specific targets and for excretion.

Predictions concerning the potential of the investigated chito-oligomers of being substrates and/or inhibitors of the P-glycoprotein (an efflux membrane transporter that is responsible for limiting cellular uptake and the distribution of xenobiotics within the human body) are not consistent between the computational tools that were used in this study. SwissADME tool predicts that all investigated COs are substrates of P-gp, but admetSAR2.0 illustrate the contrary. PreADMET tool reveals that investigated COs are not inhibitors of P-glycoprotein, but admetSAR2.0 displays that oligomers containing at least 4 monomeric units are potential inhibitors of P-glycoprotein. It illustrates that the activity of P-glycoprotein may be affected by the presence of COs and absorption and retention of COs in the cells could be impaired. This aspect must be further considered in experimental studies. Investigated COs reveal no potential to penetrate the blood brain barrier and it underlines their minimal side effects against the central nervous system. An *in vitro* study emphasized that COs with MW < 2,000 Da reflected neuroprotective effects (Santos-Moriano et al., 2018).

Investigated COs reveal no toxicity, but partially and totally deacetylated chitin oligomers are predicted to produce phospholipidosis, a disorder characterized by the accumulation in excess of phospholipids in tissues. This prediction is not unexpected as chitosan is a cationic polymer and it is known that cationic amphiphilic drugs may produce phospholipidosis (Anderson and Borlaka, 2006; Muehlbacher et al., 2012).

Literature data reflect that some xenobiotics (including drugs) are able to inhibit organic anion polypeptide transporters OATP1B1, OATP1B3, and OATP2B1. OATP1B1 and OATP1B3 are exclusively expressed in the liver, this organ being responsible for the hepatic uptake of some drugs, bile acids and some endogenous compounds. OATP2B1 is found in the liver and other tissues being associated with the oral absorption of chemicals. The inhibition of these transporters conducts to clinically relevant drug-drug interactions and to modified pharmacological effects and adverse reactions of drugs (Maeda, 2015; Alam et al., 2018). Another transporter that may be affected by the presence of xenobiotics is the organic cation transporter expressed in the kidney, OCT2. OCT2 transports compounds that are positively charged from the blood to the tubular epithelial cells, its inhibition also conducting to adverse effects (Motohashi and Inui, 2013). Consequently, predicting the inhibition of these transporters is important. Predictions obtained using admetSAR2.0 reflect that all investigated chito-oligomers are considered as possible inhibitors of the OATP1B3 and the deacetylated oligomers are also inhibitors of the OATP1B1. The inhibition of organic anion transporters OATP1B1 and OPTAP1B3 by the investigated oligomers is not an unexpected result because *in silico* models revealed the importance of lipophilicity, polarity and hydrogen bonding for OATP inhibition (Karlgren et al., 2012). Deacetylated oligomers reveal higher lipophilicity and hydrogen bonding potential that may conduct to their inhibitory effect against OATP1B1 too. Furthermore, the role of computational methods in predicting clinically relevant transporter interactions has been recognized (Türková and Zdrazil, 2019).

The outcomes of both admetSAR2.0 and Pred-hERG revealed that investigated COs, excepting the monomers, illustrate moderate potentials of hERG channel blocking. The hERG blockage potential of investigated COs increases with the molecular weight and it slightly depends on the acetylation degree and pattern. Literature data reveal the cavity of the hERG pore is large and is able to accommodate compounds with very high molecular weight (Linder et al., 2016) and that hERG inhibition positively correlates to the logP, molecular weight and rotatable bonds (Yu et al., 2016).

The hepatotoxicity of investigated COs also seems to depend on the molecular weight, the oligomers having the molecular weights between 500 and 1500 Da reflecting a weak potential of hepatotoxicity. This result is also in good correlation with published data revealing that lipophilicity and molecular weight are the most important physicochemical properties that influence the drug induced liver injury (Leeson, 2018). Furthermore, there is a slight dependence of hepatotoxicity of COs on the deacetylation degree and pattern. Literature data concerning the organ, tissue and cellular distribution COs suggest that: (i) they are usually distributed to kidney, hepatic, and splenic cells (the highest detected concentration was in hepatic cells) and lower concentrations were distributed to cardiac and lung tissues; (ii) MW and DDA influence the tissue and cellular distribution of COs and (iii) the biodegradation of COs is considered to occurring in the liver (Naveed et al., 2019).

There are some potential lacunas when predicting the pharmacokinetic profiles and toxicological endpoints of COs that might limit their effectiveness and probably affect the experimental validations. These lacunas are common to *in silico* predictions and in the case of the present study, they refer to: (i) the models used for predictions could not be adequate as the data regarding the biological activities of well-defined COs are limited and these data are not considered when building the models used for predictions; (ii) these predictions do not take into account the quantity of COs and the basic variables of experimental studies (temperature, humidity, pH, etc.); (iii) there are difficulties concerning the modeling of the toxicological endpoint because the lack of a complete understanding of its biology and of the complexity of processes involved. These lacunas may affect the experimental validations as data that were used for model building may originate from various experimental approaches that are different from those used for validation, as it is recognized that inconsistencies between predictions and experiments can often be attributed to the fact that they do not test the same assumptions (Gallion et al., 2017). In order to minimize the effects of the quality of the models built on data originated from various experimental approaches we have used well-established and recognized computational tools that are based on models obtained considering data for numerous chemical compounds and that have high predictive accuracies (higher than 70%). Furthermore, we have used both computational tools that are able to predict many pharmacological and toxicological properties, but also computational tools that are associated with a defined toxicological endpoint. The consensus of the predictions obtained using these tools increase the probability that the results are further validated in experimental tests. Due to these limitations of *in silico* predictions, the results have to be handled carefully and should not be used isolated to determine the potential hazard of COs. However, official agencies consider that the combination of computational modeling with *in vitro* testing is needed for a more efficient safety assessment of all types of chemicals.

Experimental validations of the biological effects of chemicals should be reliable, precise, and performed on a sufficiently large scale to be meaningful. These conditions may require repetitive measurement with a given assay or testing the same biological action with various assays. Consequently, in most practical cases, it is challenging to evaluate whether the available data are acceptably and complete, and to assess whether the experimental design affects the relationship with computational prediction (Gallion et al., 2017). An exhaustive experimental campaign can be time- and money-consuming also because of the intrinsic high number of variables related to the material. Focusing on chitosan, an appropriate experimental design should explore the influence of molecular weight, acetylation degree and acetylation pattern, to name a few aspects to be considered and that have been included in this study.

The potential merits of the computational predictions obtained within this study are that they highlighted some trends that relate material properties and possible side effects, which can be included in a suitable design of experiment algorithms in order to minimize the experimental efforts number and maximize the outcomes. Initially, in *vitro* basic tests through experiments that closely match the conditions used for predictions are preferably used for validation such as to avoid the complexity of the physiological pathways and possible interactions between various chemical molecules in the animal organisms. This approach could provide several advantages. First, it would lead to a structured experimental campaign, whose results can complement the fragmentary insights available in scientific literature. Secondly, experiments can be employed to assess the reliability of model predictions and thus the suitability of the chosen approach for pure predictive simulations. Thirdly, getting experimental data for well-characterized COs may provide an insight to define the applications of COs and will also drive the development of more rigorous models that also will conduct to improved predictions.

## Conclusions

The pharmacokinetic profiles of chito-oligosaccharides are rarely experimentally studied, but taking into account their promising applications, their efficacy, and safety assessment are points to be considered. Obtaining well-defined COs in terms of length, degree acetylation, and acetylation pattern are still not straightforward and computational approaches offer an advantage in such cases. Within this study, we have used various computational tools to assess the pharmacokinetic profiles and toxicological endpoints of investigated COs. Computational predictions revealed that investigated small chito-oligomers, regardless of molecular weight, acetylation degree and acetylation pattern, reflect favorable pharmacological profiles: they are not able to penetrate the blood-brain barrier, do not produce eye irritation and corrosion, reveal no mutagenicity, no carcinogenicity, and no skin sensitization potential.

As possible harmful effects we have noticed the followings: (i) all investigated COs revealed high potential of inhibition of the OATP1B3 and COs containing only deacetylated units also reflect inhibition potential of the OATP1B1; (ii) COs containing more than 2 units reflect a moderate potential of cardiotoxicity; (iii) some of considered COs reflect small probabilities to produce hepatotoxicity; (iv) smaller oligomers (*n* = 1–3) reflect a weak disruption potential against AR and GR; (v) totally deacetylated oligomers are considered to produce phospholipidosis.

Predictions concerning the interactions of investigated COs with P-glycoprotein and CYPs are unclear and they must be further considered in experimental studies.

We have also examined the influence of the molecular weight, deacetylation degree, and pattern on the pharmacological profiles and toxicological endpoints of investigated COs. The oral bioavailability of investigated COs decreases with increasing MW and deacetylation degree. Taking into account that bioavailability profile could be the main factor limiting the efficiency of a drug, this information is of interest. There is a slightly dependence of hepatotoxicity and cardiotoxicity on the molecular weight and on the deacetylation degree and pattern of COs. The cardiotoxicity of investigated COs increases poorly with the molecular weight, decreases slightly with the deacetylation degree and is not influenced by deacetylation pattern. Hepatotoxicity increases with the molecular weight, decrease with the deacetylation degree and depends on the deacetylation pattern.

## Data Availability

All datasets generated for this study are included in the manuscript/Supplementary Files.

## Author Contributions

DR performed the computations using FAF-Drugs, admetSAR, PreADMET, Pred-Skin, and Endocrine Disruptome, to results analysis and contributed to manuscript editing. MR performed computations using, SwissADME, Pred-hERG, CarcinoPred-El, and Toxtree, to results analysis and contributed to manuscript editing. CS, MS, EH, PW, TC, and GP contributed to the analysis of results, conception, and design of the manuscript. VO furnished the SMILES and structural formulas of oligomers and contributed to the conception and design of the manuscript. AI conceived and planned the study and contributed to the conception and design of the manuscript. All authors contributed to revise the manuscript and approved the submitted version.

### Conflict of Interest Statement

The authors declare that the research was conducted in the absence of any commercial or financial relationships that could be construed as a potential conflict of interest.
