# Supplementary material for: Computational Assessment of the Pharmacological Profiles of Degradation Products of Chitosan
Source: Front Bioeng Biotechnol. 2019 Sep 6;7:214. doi: 10.3389/fbioe.2019.00214 (PMC6743017; doi:10.3389/fbioe.2019.00214)
Supplement: Supplementary file 1 [file Table_1.DOCX]

Supplementary table 1. Predictions obtained using PreADMET and SwissADME tools concerning oral bioavailability of investigated chito-oligomers: MW-molecular weight, HBA – hydrogen bond acceptors, HBD- hydrogen bonds donors, LogP – partition coefficient, TPSA – topological polar surface area, Rotors – number of rotatable bonds. Green cells correspond to compounds having a good oral bioavailability, yellow cells to compound with a mean oral bioavailability and red cells to compound with low oral bioavailability.

| **Compound/**  **Tool** | **PreADMET** | **SwissADME** | | | | | |
| --- | --- | --- | --- | --- | --- | --- | --- |
|  | **Lipinski rule** | **Lipinski rule** | **Ghose rule** | **Veber rule** | **Egan rule** | **Muegge rule** | **Bioavailability score** |
| A | Yes; 0 violation | Yes; 0 violation | No; 1 violation: LOGP<-0.4 | Yes | Yes | Yes | 0.55 |
| 2A | No; 2 violations: HBA>10, HBD>5 | No; 2 violations: HBA>10, HBD>5 | No; 1 violation: LOGP<-0.4 | No; 1 violation: TPSA>140 | No; 1 violation: TPSA>131.6 | No; 4 violations: LOGP<-2, TPSA>150, HBA>10, HBD>5 | 0.17 |
| 3A | No; 3 violations: MW>500, HBA>10, HBD>5 | No; 3 violations: MW>500, HBA>10, HBD>5 | No; 4 violations: MW>480, LOGP<-0.4, MR>130, #atoms>70 | No; 2 violations: Rotors>10, TPSA>140 | No; 1 violation: TPSA>131.6 | No; 5 violations: MW>600, LOGP<-2, TPSA>150, HBA>10, HBD>5 | 0.17 |
| 4A | No; 3 violations: MW>500, HBA>10, HBD>5 | No; 3 violations: MW>500, HBA>10, HBD>5 | No; 4 violations: MW>480, LOGP<-0.4, MR>130, #atoms>70 | No; 2 violations: Rotors>10, TPSA>140 | No; 1 violation: TPSA>131.6 | No; 6 violations: MW>600, LOGP<-2, TPSA>150, Rotors>15, HBA>10, HBD>5 | 0.17 |
| 5A | No; 3 violations: MW>500, HBA>10, HBD>5 | No; 3 violations: MW>500, HBA>10, HBD>5 | No; 4 violations: MW>480, LOGP<-0.4, MR>130, #atoms>70 | No; 2 violations: Rotors>10, TPSA>140 | No; 1 violation: TPSA>131.6 | No; 6 violations: MW>600, LOGP<-2, TPSA>150, Rotors>15, HBA>10, HBD>5 | 0.17 |
| 6A | No; 3 violations: MW>500, HBA>10, HBD>5 | No; 3 violations: MW>500, HBA>10, HBD>5 | No; 4 violations: MW>480, LOGP<-0.4, MR>130, #atoms>70 | No; 2 violations: Rotors>10, TPSA>140 | No; 1 violation: TPSA>131.6 | No; 6 violations: MW>600, LOGP<-2, TPSA>150, Rotors>15, HBA>10, HBD>5 | 0.17 |
| 8A | No; 3 violations: MW>500, HBA>10, HBD>5 | No; 3 violations: MW>500, HBA>10, HBD>5 | No; 4 violations: MW>480, LOGP<-0.4, MR>130, #atoms>70 | No; 2 violations: Rotors>10, TPSA>140 | No; 1 violation: TPSA>131.6 | No; 7 violations: MW>600, LOGP<-2, TPSA>150, #rings>7, Rotors>15, HBA>10, HBD>5 | 0.17 |
| ADA | No; 3 violations: MW>500, HBA>10, HBD>5 | No; 3 violations: MW>500, HBA>10, HBD>5 | No; 3 violations: MW>480, LOGP<-0.4, #atoms>70 | No; 2 violations: Rotors>10, TPSA>140 | No; 1 violation: TPSA>131.6 | No; 4 violations: LOGP<-2, TPSA>150, HBA>10, HBD>5 | 0.17 |
| DA | No; 2 violations: HBA>10, HBD>5 | No; 2 violations: HBA>10, HBD>5 | No; 1 violation: LOGP<-0.4 | No; 1 violation: TPSA>140 | No; 1 violation: TPSA>131.6 | No; 3 violations: LOGP<-2, TPSA>150, HBD>5 | 0.17 |
| DADA | No; 3 violations: MW>500, HBA>10, HBD>5 | No; 3 violations: MW>500, HBA>10, HBD>5 | No; 4 violations: MW>480, LOGP<-0.4, MR>130, #atoms>70 | No; 2 violations: Rotors>10, TPSA>140 | No; 1 violation: TPSA>131.6 | No; 5 violations: MW>600, LOGP<-2, TPSA>150, HBA>10, HBD>5 | 0.17 |
| ADAD | No; 3 violations: MW>500, HBA>10, HBD>5 | No; 3 violations: MW>500, HBA>10, HBD>5 | No; 4 violations: MW>480, LOGP<-0.4, MR>130, #atoms>70 | No; 2 violations: Rotors>10, TPSA>140 | No; 1 violation: TPSA>131.6 | No; 5 violations: MW>600, LOGP<-2, TPSA>150, HBA>10, HBD>5 | 0.17 |
| AADD | No; 3 violations: MW>500, HBA>10, HBD>5 | No; 3 violations: MW>500, HBA>10, HBD>5 | No; 4 violations: MW>480, LOGP<-0.4, MR>130, #atoms>70 | No; 2 violations: Rotors>10, TPSA>140 | No; 1 violation: TPSA>131.6 | No; 5 violations: MW>600, LOGP<-2, TPSA>150, HBA>10, HBD>5 | 0.17 |
| DDAA | No; 3 violations: MW>500, HBA>10, HBD>5 | No; 3 violations: MW>500, HBA>10, HBD>5 | No; 4 violations: MW>480, LOGP<-0.4, MR>130, #atoms>70 | No; 2 violations: Rotors>10, TPSA>140 | No; 1 violation: TPSA>131.6 | No; 5 violations: MW>600, LOGP<-2, TPSA>150, HBA>10, HBD>5 | 0.17 |
| DAAD | No; 3 violations: MW>500, HBA>10, HBD>5 | No; 3 violations: MW>500, HBA>10, HBD>5 | No; 4 violations: MW>480, LOGP<-0.4, MR>130, #atoms>70 | No; 2 violations: Rotors>10, TPSA>140 | No; 1 violation: TPSA>131.6 | No; 5 violations: MW>600, LOGP<-2, TPSA>150, HBA>10, HBD>5 | 0.17 |
| ADDA | No; 3 violations: MW>500, HBA>10, HBD>5 | No; 3 violations: MW>500, HBA>10, HBD>5 | No; 4 violations: MW>480, LOGP<-0.4, MR>130, #atoms>70 | No; 2 violations: Rotors>10, TPSA>140 | No; 1 violation: TPSA>131.6 | No; 5 violations: MW>600, LOGP<-2, TPSA>150, HBA>10, HBD>5 | 0.17 |
| DADADA | No; 3 violations: MW>500, HBA>10, HBD>5 | No; 3 violations: MW>500, HBA>10, HBD>5 | No; 4 violations: MW>480, LOGP<-0.4, MR>130, #atoms>70 | No; 2 violations: Rotors>10, TPSA>140 | No; 1 violation: TPSA>131.6 | No; 6 violations: MW>600, LOGP<-2, TPSA>150, Rotors>15, HBA>10, HBD>5 | 0.17 |
| ADADAD | No; 3 violations: MW>500, HBA>10, HBD>5 | No; 3 violations: MW>500, HBA>10, HBD>5 | No; 4 violations: MW>480, LOGP<-0.4, MR>130, #atoms>70 | No; 2 violations: Rotors>10, TPSA>140 | No; 1 violation: TPSA>131.6 | No; 6 violations: MW>600, LOGP<-2, TPSA>150, Rotors>15, HBA>10, HBD>5 | 0.17 |
| DADADADA | No; 3 violations: MW>500, HBA>10, HBD>5 | No; 3 violations: MW>500, HBA>10, HBD>5 | No; 4 violations: MW>480, LOGP<-0.4, MR>130, #atoms>70 | No; 2 violations: Rotors>10, TPSA>140 | No; 1 violation: TPSA>131.6 | No; 7 violations: MW>600, LOGP<-2, TPSA>150, #rings>7, Rotors>15, HBA>10, HBD>5 | 0.17 |
| DDA | No; 3 violations: MW>500, HBA>10, HBD>5 | No; 3 violations: MW>500, HBA>10, HBD>5 | No; 3 violations: MW>480, LOGP<-0.4, #atoms>70 | No; 1 violation: TPSA>140 | No; 1 violation: TPSA>131.6 | No; 4 violations: LOGP<-2, TPSA>150, HBA>10, HBD>5 | 0.17 |
| ADDDAD | No; 3 violations: MW>500, HBA>10, HBD>5 | No; 3 violations: MW>500, HBA>10, HBD>5 | No; 4 violations: MW>480, LOGP<-0.4, MR>130, #atoms>70 | No; 2 violations: Rotors>10, TPSA>140 | No; 1 violation: TPSA>131.6 | No; 6 violations: MW>600, LOGP<-2, TPSA>150, Rotors>15, HBA>10, HBD>5 | 0.17 |
| DDDADA | No; 3 violations: MW>500, HBA>10, HBD>5 | No; 3 violations: MW>500, HBA>10, HBD>5 | No; 4 violations: MW>480, LOGP<-0.4, MR>130, #atoms>70 | No; 2 violations: Rotors>10, TPSA>140 | No; 1 violation: TPSA>131.6 | No; 6 violations: MW>600, LOGP<-2, TPSA>150, Rotors>15, HBA>10, HBD>5 | 0.17 |
| D | Yes; 0 violation | Yes; 0 violation | No; 2 violations: LOGP<-0.4, MR<40 | Yes | Yes | No; 2 violations: MW<200, LOGP<-2 | 0.55 |
| 2D | No; 2 violations: HBA>10, HBD>5 | No; 2 violations: HBA>10, HBD>5 | No; 1 violation: LOGP<-0.4 | No; 1 violation: TPSA>140 | No; 1 violation: TPSA>131.6 | No; 3 violations: LOGP<-2, TPSA>150, HBD>5 | 0.17 |
| 3D | No; 3 violations: MW>500, HBA>10, HBD>5 | No; 3 violations: MW>500, HBA>10, HBD>5 | No; 3 violations: MW>480, LOGP<-0.4, #atoms>70 | No; 1 violation: TPSA>140 | No; 1 violation: TPSA>131.6 | No; 4 violations: LOGP<-2, TPSA>150, HBA>10, HBD>5 | 0.17 |
| 4D | No; 3 violations: MW>500, HBA>10, HBD>5 | No; 3 violations: MW>500, HBA>10, HBD>5 | No; 4 violations: MW>480, LOGP<-0.4, MR>130, #atoms>70 | No; 1 violation: TPSA>140 | No; 1 violation: TPSA>131.6 | No; 5 violations: MW>600, LOGP<-2, TPSA>150, HBA>10, HBD>5 | 0.17 |
| 5D | No; 3 violations: MW>500, HBA>10, HBD>5 | No; 3 violations: MW>500, HBA>10, HBD>5 | No; 4 violations: MW>480, LOGP<-0.4, MR>130, #atoms>70 | No; 2 violations: Rotors>10, TPSA>140 | No; 1 violation: TPSA>131.6 | No; 5 violations: MW>600, LOGP<-2, TPSA>150, HBA>10, HBD>5 | 0.17 |
| 6D | No; 3 violations: MW>500, HBA>10, HBD>5 | No; 3 violations: MW>500, HBA>10, HBD>5 | No; 4 violations: MW>480, LOGP<-0.4, MR>130, #atoms>70 | No; 2 violations: Rotors>10, TPSA>140 | No; 1 violation: TPSA>131.6 | No; 6 violations: MW>600, LOGP<-2, TPSA>150, Rotors>15, HBA>10, HBD>5 | 0.17 |
| 8D | No; 3 violations: MW>500, HBA>10, HBD>5 | No; 3 violations: MW>500, HBA>10, HBD>5 | No; 4 violations: MW>480, LOGP<-0.4, MR>130, #atoms>70 | No; 2 violations: Rotors>10, TPSA>140 | No; 1 violation: TPSA>131.6 | No; 7 violations: MW>600, LOGP<-2, TPSA>150, #rings>7, Rotors>15, HBA>10, HBD>5 | 0.17 |
