# Supplementary material for: Computational Assessment of the Pharmacological Profiles of Degradation Products of Chitosan
Source: Front Bioeng Biotechnol. 2019 Sep 6;7:214. doi: 10.3389/fbioe.2019.00214 (PMC6743017; doi:10.3389/fbioe.2019.00214)
Supplement: Supplementary file 2 [file Table_2.DOCX]

Supplementary table 2. Predictions obtained using PreADMET tool concerning the pharmacokinetics profile of investigated chito-oligomers: HIA– human intestinal absorption, BBB – blood brain barrier permeation, PPB – percentage of chemical molecule bound to plasma proteins, P-gp – P glycoprotein, CYP – human cytochrome P450, logKp – skin permeation coefficient. HIA takes values between 0 and 100: HIA<20% reflects poor absorption, 20%<HIA<70% corresponds to compounds reflecting mean absorption, 70%<HIA<100% correspond to a high intestinal absorption. BBB takes values from 0 to 100: BBB<0.1 illustrates low absorption in the central nervous system (CNS), 1<BBB<2 reflects middle absorption in CNS and BBB>2 indicates high absorption in CNS. PPB takes values between 0 and 100 and PPB>90% indicates a strong binding energy and PPB<90% indicates low binding energy with plasma proteins.

| **Compound** | **HIA (%)** | **BBB**  **(%)** | **PPB**  **(%)** | **P-gp inhibition** | **CYP2C19 inhibitor** | **CYP2C9 inhibitor** | **CYP2D6 inhibitor** | **CYP3A4 inhibitor** | **CYP2D6**  **substrate** | **CYP3A4 substrate** | **LogKp**  **(cm/hour)** |
| --- | --- | --- | --- | --- | --- | --- | --- | --- | --- | --- | --- |
| A | 22.29 | 0.064 | 4.138 | Non | Non | Non | Inhibitor | Non | Non | Weakly | -5.45 |
| 2A | 2.97 | 0.029 | 6.068 | Non | Non | Non | Non | Inhibitor | Non | Weakly | -5.17 |
| 3A | 0.24 | 0.028 | 4.570 | Non | Non | Non | Non | Inhibitor | Non | Weakly | -5.20 |
| 4A | 0 | 0.028 | 2.878 | Non | Non | Non | Non | Inhibitor | Non | Weakly | -5.14 |
| 5A | 0 | 0.029 | 0 | Non | Non | Non | Non | Inhibitor | Non | Weakly | -4.83 |
| 6A | 0 | 0.031 | 0 | Non | Non | Non | Non | Inhibitor | Non | Substrate | -3.77 |
| 8A | 0 | 0.037 | 0 | Non | Non | Non | Non | Inhibitor | Non | Weakly | -2.31 |
| ADA | 0 | 0.027 | 39.691 | Non | Non | Non | Inhibitor | Inhibitor | Non | Weakly | -5.25 |
| DA | 1.90 | 0.028 | 35.987 | Non | Non | Non | Inhibitor | Inhibitor | Weakly | Weakly | -5.26 |
| DADA | 0 | 0.027 | 42.984 | Non | Non | Non | Inhibitor | Inhibitor | Non | Weakly | -5.26 |
| ADAD | 0 | 0.027 | 42.998 | Non | Non | Non | Inhibitor | Inhibitor | Non | Substrate | -5.26 |
| AADD | 0 | 0.027 | 42.998 | Non | Non | Non | Inhibitor | Inhibitor | Non | Substrate | -5.26 |
| DDAA | 0 | 0.027 | 42.984 | Non | Non | Non | Inhibitor | Inhibitor | Non | Weakly | -5.26 |
| DAAD | 0 | 0.027 | 43.007 | Non | Non | Non | Inhibitor | Inhibitor | Non | Substrate | -5.26 |
| ADDA | 0 | 0.027 | 42.973 | Non | Non | Non | Inhibitor | Inhibitor | Non | Weakly | -5.26 |
| DADADA | 0 | 0.027 | 45.305 | Non | Non | Non | Inhibitor | Inhibitor | Non | Substrate | -4.94 |
| ADADAD | 0 | 0.027 | 45.308 | Non | Non | Non | Inhibitor | Inhibitor | Non | Substrate | -4.94 |
| DADADADA | 0 | 0.027 | 46.080 | Non | Non | Non | Inhibitor | Inhibitor | Non | Substrate | -2.68 |
| DDA | 0 | 0.027 | 37.072 | Non | Non | Non | Inhibitor | Inhibitor | Substrate | Weakly | -5.28 |
| ADDDAD | 0 | 0.027 | 46.706 | Non | Non | Non | Inhibitor | Inhibitor | Weakly | Substrate | -5.12 |
| DDDADA | 0 | 0.027 | 46.697 | Non | Non | Non | Inhibitor | Inhibitor | Weakly | Substrate | -5.12 |
| D | 60.25 | 0.039 | 27.804 | Non | Non | Inhibitor | Inhibitor | Non | Weakly | Weakly | -5.51 |
| 2D | 6.01 | 0.028 | 22.054 | Non | Non | Non | Inhibitor | Inhibitor | Substrate | Weakly | -5.57 |
| 3D | 0 | 0.027 | 25.535 | Non | Non | Non | Inhibitor | Inhibitor | Substrate | Weakly | -5.31 |
| 4D | 0 | 0.027 | 36.474 | Non | Non | Non | Inhibitor | Inhibitor | Substrate | Weakly | -5.32 |
| 5D | 0 | 0.027 | 65.386 | Non | Non | Non | Inhibitor | Inhibitor | Substrate | Substrate | -5.31 |
| 6D | 0 | 0.027 | 93.384 | Non | Non | Non | Inhibitor | Inhibitor | Substrate | Substrate | -5.28 |
| 8D | Too big to be computed | | | | | | | | | | |
