# Supplementary material for: Computational Assessment of the Pharmacological Profiles of Degradation Products of Chitosan
Source: Front Bioeng Biotechnol. 2019 Sep 6;7:214. doi: 10.3389/fbioe.2019.00214 (PMC6743017; doi:10.3389/fbioe.2019.00214)
Supplement: Supplementary file 3 [file Table_3.docx]

Supplementary table 3. Predictions obtained using SwissADME tool concerning the pharmacokinetics profile of investigated chito-oligomers: GI – gastrointestinal absorbíon, BBB – blood brain barrier permeation, P-gp – P glycoprotein, CYP – human cytochrome P450, logKp – skin permeation coefficient.

| **Chito-oligomer** | **GI** | **BBB** | **P-gp substrate** | **CYP1A2 inhibitor** | **CYP2C19 inhibitor** | **CYP2C9 inhibitor** | **CYP2D6 inhibitor** | **CYP3A4 inhibitor** | **LogKp**  **(cm/s)** |
| --- | --- | --- | --- | --- | --- | --- | --- | --- | --- |
| A | Low | No | Yes | No | No | No | No | No | -8.47 |
| 2A | Low | No | Yes | No | No | No | No | No | -12.25 |
| 3A | Low | No | Yes | No | No | No | No | No | -15.01 |
| 4A | Low | No | Yes | No | No | No | No | No | -17.78 |
| 5A | Low | No | Yes | No | No | No | No | No | -20.55 |
| 6A | Low | No | Yes | No | No | No | No | No | -23.30 |
| 8A | Low | No | Yes | No | No | No | No | No | -28.84 |
| ADA | Low | No | Yes | No | No | No | No | No | -14.95 |
| DA | Low | No | Yes | No | No | No | No | No | -12.18 |
| DADA | Low | No | Yes | No | No | No | No | No | -17.64 |
| ADAD | Low | No | Yes | No | No | No | No | No | -17.64 |
| AADD | Low | No | Yes | No | No | No | No | No | -17.64 |
| DDAA | Low | No | Yes | No | No | No | No | No | -17.64 |
| DAAD | Low | No | Yes | No | No | No | No | No | -17.64 |
| ADDA | Low | No | Yes | No | No | No | No | No | -17.64 |
| DADADA | Low | No | Yes | No | No | No | No | No | -23.10 |
| ADADAD | Low | No | Yes | No | No | No | No | No | -23.10 |
| DADADADA | Low | No | Yes | No | No | No | No | No | -28.56 |
| DDA | Low | No | Yes | No | No | No | No | No | -14.87 |
| ADDDAD | Low | No | Yes | No | No | No | No | No | -23.03 |
| DDDADA | Low | No | Yes | No | No | No | No | No | -23.03 |
| D | Low | No | Yes | No | No | No | No | No | -9.88 |
| 2D | Low | No | Yes | No | No | No | No | No | -12.12 |
| 3D | Low | No | Yes | No | No | No | No | No | -14.81 |
| 4D | Low | No | Yes | No | No | No | No | No | -17.50 |
| 5D | Low | No | Yes | No | No | No | No | No | -20.20 |
| 6D | Low | No | Yes | No | No | No | No | No | -22.89 |
| 8D | Low | No | Yes | No | No | No | No | No | -28.28 |
