# Supplementary material for: Computational Assessment of the Pharmacological Profiles of Degradation Products of Chitosan
Source: Front Bioeng Biotechnol. 2019 Sep 6;7:214. doi: 10.3389/fbioe.2019.00214 (PMC6743017; doi:10.3389/fbioe.2019.00214)
Supplement: Supplementary file 4 [file Table_4.DOCX]

Supplementary table 4. Predictions obtained using admetSAR2.0 tool concerning the probability that investigated chito-oligomers are substrates/ inhibitors of the human cytochromes (CYP) involved in the xenobiotics metabolism.

| **Compound** | **CYP3A4**  **non substrate** | **CYP2C9**  **non substrate** | **CYPSD6**  **non substrate** | **CYP3A4i**  **non inhibitor** | **CYP2C9**  **non inhibitor** | **CYP2C19**  **non inhibitor** | **CYP2D6i**  **non inhibitor** | **CYP1A2**  **non inhibitor** |
| --- | --- | --- | --- | --- | --- | --- | --- | --- |
| A | 0.58 | 0.81 | 0.87 | 0.99 | 0.95 | 0.94 | 0.97 | 0.97 |
| 2A | 0.55 | 0.81 | 0.87 | 0.92 | 0.92 | 0.92 | 0.94 | 0.96 |
| 3A | 0.55 | 0.81 | 0.97 | 0.86 | 0.92 | 0.93 | 0.93 | 0.96 |
| 4A | 0.55 | 0.81 | 0.87 | 0.86 | 0.92 | 0.93 | 0.93 | 0.96 |
| 5A | 0.55 | 0.81 | 0.87 | 0.86 | 0.92 | 0.93 | 0.93 | 0.96 |
| 6A | 0.55 | 0.81 | 0.87 | 0.86 | 0.92 | 0.93 | 0.93 | 0.96 |
| 8A | 0.55 | 0.81 | 0.87 | 0.86 | 0.92 | 0.93 | 0.93 | 0.96 |
| ADA | 0.57 | 1.00 | 0.85 | 0.94 | 0.91 | 0.90 | 0.93 | 0.94 |
| DA | 0.56 | 1.00 | 0.85 | 0.96 | 0.89 | 0.89 | 0.91 | 0.92 |
| DADA | 0.58 | 1.00 | 0.85 | 0.95 | 0.93 | 0.91 | 0.93 | 0.94 |
| ADAD | 0.57 | 1.00 | 0.85 | 0.95 | 0.91 | 0.91 | 0.93 | 0.95 |
| AADD | 0.57 | 1.00 | 0.85 | 0.95 | 0.91 | 0.91 | 0.93 | 0.95 |
| DDAA | 0.58 | 1.00 | 0.85 | 0.95 | 0.91 | 0.91 | 0.93 | 0.95 |
| DAAD | 0.58 | 1.00 | 0.85 | 0.95 | 0.91 | 0.91 | 0.93 | 0.95 |
| ADDA | 0.57 | 1.00 | 0.85 | 0.94 | 0.91 | 0.91 | 0.93 | 0.95 |
| DADADA | 0.58 | 1.00 | 0.85 | 0.92 | 0.91 | 0.92 | 0.92 | 0.94 |
| ADADAD | 0.57 | 1.00 | 0.85 | 0.91 | 0.91 | 0.92 | 0.92 | 0.96 |
| DADADADA | 0.58 | 1.00 | 0.85 | 0.92 | 0.91 | 0.92 | 0.92 | 0.95 |
| DDA | 0.58 | 1.00 | 0.85 | 0.96 | 0.89 | 0.88 | 0.91 | 0.92 |
| ADDDAD | 0.57 | 1.00 | 0.85 | 0.95 | 0.91 | 0.91 | 0.93 | 0.95 |
| DDDADA | 0.58 | 1.00 | 0.85 | 0.94 | 0.91 | 0.90 | 0.93 | 0.95 |
| D | 0.66 | 1.00 | 0.32 | 0.98 | 0.93 | 0.92 | 0.94 | 0.94 |
| 2D | 0.52 | 1.00 | 0.82 | 0.98 | 0.88 | 0.86 | 0.89 | 0.89 |
| 3D | 0.52 | 1.00 | 0.83 | 0.98 | 0.89 | 0.87 | 0.89 | 0.90 |
| 4D | 0.52 | 1.00 | 0.83 | 0.98 | 0.89 | 0.87 | 0.89 | 0.90 |
| 5D | 0.51 | 1.00 | 0.82 | 0.98 | 0.89 | 0.87 | 0.89 | 0.90 |
| 6D | 0.52 | 1.00 | 0.83 | 0.98 | 0.89 | 0.87 | 0.89 | 0.90 |
| 8D | 0.52 | 1.00 | 0.83 | 0.98 | 0.89 | 0.87 | 0.89 | 0.90 |
