# Supplementary material for: Computational Assessment of the Pharmacological Profiles of Degradation Products of Chitosan
Source: Front Bioeng Biotechnol. 2019 Sep 6;7:214. doi: 10.3389/fbioe.2019.00214 (PMC6743017; doi:10.3389/fbioe.2019.00214)
Supplement: Supplementary file 5 [file Table_5.DOCX]

Supplementary table 5. Predictions concerning carcinogenic and mutagenic potential of considered COs.

| **Compound/ tool** | **Toxtree** | | | **Carcino**  **PredEl** |
| --- | --- | --- | --- | --- |
|  | **Non-genotoxic carcinogenicity** | **Genotoxic carcinogenicity** | **AMES toxic** | **carcinogen** |
| A | No | No | No | No |
| 2A | No | No | No | No |
| 3A | No | No | No | No |
| 4A | No | No | No | No |
| 5A | No | No | No | No |
| 6A | No | No | No | No |
| 8A | No | No | No | No |
| ADA | No | No | No | No |
| DA | No | No | No | No |
| DADA | No | No | No | No |
| ADAD | No | No | No | No |
| AADD | No | No | No | No |
| DDAA | No | No | No | No |
| DAAD | No | No | No | No |
| ADDA | No | No | No | No |
| DADADA | No | No | No | No |
| ADADAD | No | No | No | No |
| DADADADA | No | No | No | No |
| DDA | No | No | No | No |
| ADDDAD | No | No | No | No |
| DDDADA | No | No | No | No |
| D | No | No | No | No |
| 2D | No | No | No | No |
| 3D | No | No | No | No |
| 4D | No | No | No | No |
| 5D | No | No | No | No |
| 6D | No | No | No | No |
| 8D | No | No | No | No |
