# Supplementary material for: Computational Assessment of the Pharmacological Profiles of Degradation Products of Chitosan
Source: Front Bioeng Biotechnol. 2019 Sep 6;7:214. doi: 10.3389/fbioe.2019.00214 (PMC6743017; doi:10.3389/fbioe.2019.00214)
Supplement: Supplementary file 6 [file Table_6.DOCX]

Supplementary table 6. Predictions concerning the skin penetration and skin sensitization potential of investigated oligomers. Numbers in the table reflect the accuracy of every prediction.

| **Compound/ Computational tool** | **PredSkin** | | | | | |
| --- | --- | --- | --- | --- | --- | --- |
|  | **Human skin sensitization** | **Murine local lymph node assay (LLNA)** | **Direct peptide reactivity assay (DPRA)** | **Human cell line activation test (h-CLAT)** | **KeratinoSens**  **TM** | **Consensus** |
| A | Sensitizer 60% | Non-sensitizer 90% | Sensitizer  50% | Sensitizer 70% | Non-sensitizer 80% | Sensitizer 70% |
| 2A | Sensitizer 60% | Non-sensitizer 90% | Sensitizer  50% | Non-sensitizer 60% | Non-sensitizer 70% | Non-sensitizer 60% |
| 3A | Sensitizer 60% | Non-sensitizer 90% | Sensitizer  50% | Non-sensitizer 60% | Non-sensitizer 70% | Non-sensitizer 60% |
| 4A | Sensitizer 60% | Non-sensitizer 90% | Sensitizer  50% | Non-sensitizer 60% | Non-sensitizer 70% | Non-sensitizer 60% |
| 5A | Sensitizer 60% | Non-sensitizer 90% | Sensitizer  50% | Non-sensitizer 60% | Non-sensitizer 70% | Non-sensitizer 60% |
| 6A | Sensitizer 60% | Non-sensitizer 90% | Sensitizer  50% | Non-sensitizer 60% | Non-sensitizer 70% | Non-sensitizer 60% |
| 8A | Sensitizer 60% | Non-sensitizer 90% | Sensitizer  50% | Non-sensitizer 60% | Non-sensitizer 70% | Non-sensitizer 60% |
| ADA | Sensitizer 60% | Non-sensitizer 50% | Non-sensitizer  50% | Non-sensitizer 60% | Non-sensitizer 70% | Non-sensitizer 60% |
| DA | Sensitizer 60% | Non-sensitizer 80% | Non-sensitizer  50% | Non-sensitizer 60% | Non-sensitizer 60% | Non-sensitizer 70% |
| DADA | Sensitizer 60% | Non-sensitizer 50% | Non-sensitizer  80% | Non-sensitizer 60% | Non-sensitizer 70% | Non-sensitizer 60% |
| AADD | Sensitizer 60% | Non-sensitizer 50% | Non-sensitizer  50% | Non-sensitizer 60% | Non-sensitizer 70% | Non-sensitizer 60% |
| DAAD | Sensitizer 60% | Non-sensitizer 50% | Non-sensitizer  50% | Non-sensitizer 60% | Non-sensitizer 70% | Non-sensitizer 60% |
| DDAA | Sensitizer 60% | Non-sensitizer 50% | Non-sensitizer  50% | Non-sensitizer 60% | Non-sensitizer 70% | Non-sensitizer 60% |
| ADDA | Sensitizer 60% | Non-sensitizer 50% | Non-sensitizer  50% | Non-sensitizer 60% | Non-sensitizer 70% | Non-sensitizer 60% |
| DADADA | Sensitizer 60% | Non-sensitizer 50% | Non-sensitizer  50% | Non-sensitizer 60% | Non-sensitizer 70% | Non-sensitizer 60% |
| ADADAD | Sensitizer 60% | Non-sensitizer 50% | Non-sensitizer  50% | Non-sensitizer 60% | Non-sensitizer 70% | Non-sensitizer 60% |
| DADADADA | Sensitizer 60% | Non-sensitizer 50% | Non-sensitizer  50% | Non-sensitizer 60% | Non-sensitizer 70% | Non-sensitizer 60% |
| DDA | Sensitizer 60% | Non-sensitizer 50% | Non-sensitizer  50% | Non-sensitizer 60% | Non-sensitizer 70% | Non-sensitizer 60% |
| ADDDAD | Sensitizer 60% | Non-sensitizer 50% | Non-sensitizer  50% | Non-sensitizer 60% | Non-sensitizer 70% | Non-sensitizer 60% |
| DDDADA | Sensitizer 60% | Non-sensitizer 50% | Non-sensitizer  50% | Non-sensitizer 60% | Non-sensitizer 70% | Non-sensitizer 60% |
| D | Sensitizer 80% | Non-sensitizer 90% | Non-sensitizer  60% | Sensitizer 70% | Non-sensitizer 90% | Sensitizer 60% |
| 2D | Sensitizer 80% | Non-sensitizer 90% | Non-sensitizer 60% | Non-sensitizer 60% | Non-sensitizer 80% | Non-sensitizer 70% |
| 3D | Sensitizer 80% | Non-sensitizer 90% | Non-sensitizer 60% | Non-sensitizer 60% | Non-sensitizer 80% | Non-sensitizer 70% |
| 4D | Sensitizer 80% | Non-sensitizer 90% | Non-sensitizer 60% | Non-sensitizer 60% | Non-sensitizer 80% | Non-sensitizer 70% |
| 5D | Sensitizer 80% | Non-sensitizer 90% | Non-sensitizer 60% | Non-sensitizer 60% | Non-sensitizer 80% | Non-sensitizer 70% |
| 6D | Sensitizer 80% | Non-sensitizer 90% | Non-sensitizer 60% | Non-sensitizer 60% | Non-sensitizer 80% | Non-sensitizer 70% |
| 8D | Sensitizer 80% | Non-sensitizer 90% | Non-sensitizer 60% | Non-sensitizer 60% | Non-sensitizer 80% | Non-sensitizer 70% |
